# Supplementary material for: Calcium-based multi-element chemistry for grid-scale electrochemical energy storage
Source: Nat Commun. 2016 Mar 22;7:10999. doi: 10.1038/ncomms10999 (PMC4804165; doi:10.1038/ncomms10999)
Supplement: Supplementary Information — Supplementary Figure 1, Supplementary Tables 1-7, Supplementary Note 1 and Supplementary Reference [file ncomms10999-s1.pdf]

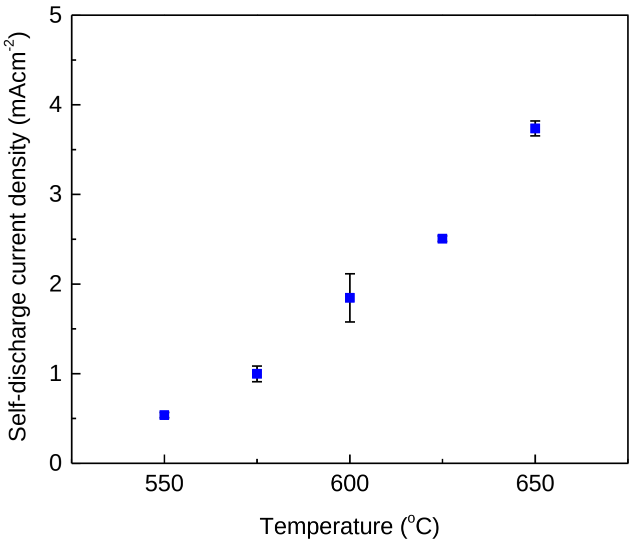

Supplementary Figure 1 | Temperature dependence of self-discharge current density of Ca-Mg(90-10 mol%)|LiCl-$CaCl_2$|Bi cell. Measurements were carried out at 1.1 V. Error bars of plots were defined by s.d.

Supplementary Table 1 | Self-discharge current density of cells with Bi positive electrode at 1.25 V.

Negative electrode	Salt	Temperature (°C)	j_{self} (mA cm^{-2})
Ca	$CaCl_2$	800	> 1000
Ca	$CaCl_2$-CaI (51.4-48.6 mol%)	650	> 300
Ca	LiCl-$CaCl_2$ (65-35 mol%)	650	~10
Ca-Mg (20-80 mol%)	LiCl-$CaCl_2$ (65-35 mol%)	650	~1
Ca-Mg (20-80 mol%)	LiCl-KCl-$CaCl_2$ (37-28-35 mol%)	650	~4
Ca-Mg (20-80 mol%)	KCl-$CaCl_2$ (74-26 mol%)	650	~10
Ca-Mg (20-80 mol%)	LiCl-NaCl-$CaCl_2$ (37-28-35 mol%)	650	~12
Ca-Mg (90-10 mol%)	LiCl-$CaCl_2$ (65-35mol%)	650	~4

Supplementary Table 2 | Capacity and energy density per volume of electrodes of Ca-Mg||Bi cell in Fig. 1b. The composition of the Ca in the negative electrode was 20 mol%. Operating temperature and current density were 650 °C and 200 mA cm^{-2}, respectively.

	Ca	Mg	Bi	Total
Mass / g	0.397	0.964	6.00	7.36
Density / g cm^{-3}	1.55	1.74	9.78	
Volume / cm^{3}	0.256	0.554	0.613	1.42
Atomic mass / g mol^{-1}	40.08	24.31	208.98	
Amount of substance / mol	9.91E-03	3.97E-02	2.87E-02	
Theoretical discharge capacity / Ah				0.569
Achieved discharge capacity / Ah				0.539
Achieved discharge energy / Wh				0.280
Gravimetric discharge capacity density / Ah kg^{-1}				73.2
Volumetric discharge capacity density / Ah L^{-1}				379
Gravimetric discharge energy density / Wh kg^{-1}				38.0
Volumetric discharge energy density / Wh L^{-1}				197

Supplementary Table 3 | Capacity and energy density per volume of electrodes of Ca-Mg||Bi cell in Fig. 1b. The composition of the Ca in the negative electrode was 90 mol%. Operating temperature and current density were 650 °C and 200 mA cm^{-2}, respectively.

	Ca	Mg	Bi	Total
Mass / g	0.899	0.061	14.00	14.96
Density / g cm^{-3}	1.55	1.74	9.78	
Volume / cm^{3}	0.580	0.035	1.43	2.05
Atomic mass / g mol^{-1}	40.08	24.31	208.98	
Amount of substance / mol	2.24E-02	2.51E-03	6.70E-02	
Theoretical discharge capacity / Ah				1.33
Achieved discharge capacity / Ah				0.952
Achieved discharge energy / Wh				0.673
Gravimetric discharge capacity density / Ah kg^{-1}				63.6
Volumetric discharge capacity density / Ah L^{-1}				465
Gravimetric discharge energy density / Wh kg^{-1}				45.0
Volumetric discharge energy density / Wh L^{-1}				329

Supplementary Table 4 | Capacity and energy density per volume of electrodes of Ca-Mg||Sb cell in Fig. 1b. The composition of the Ca in the negative electrode was 90 mol%. Operating temperature and current density were 650 °C and 200 mA cm^{-2}, respectively.

	Ca	Mg	Sb	Total
Mass / g	1.173	0.079	8.19	9.44
Density / g cm^{-3}	1.55	1.74	6.70	
Volume / cm^{3}	0.757	0.045	1.22	2.03
Atomic mass / g mol^{-1}	40.08	24.31	121.76	
Amount of substance / mol	2.93E-02	3.25E-03	6.73E-02	
Theoretical discharge capacity / Ah				1.08
Achieved discharge capacity / Ah				0.881
Achieved discharge energy / Wh				0.778
Gravimetric discharge capacity density / Ah kg^{-1}				93.3
Volumetric discharge capacity density / Ah L^{-1}				435
Gravimetric discharge energy density / Wh kg^{-1}				82.4
Volumetric discharge energy density / Wh L^{-1}				384

Supplementary Table 5 | Capacity and energy density per volume of electrodes of Ca-Mg||Bi cell in Fig. 2. The composition of the Ca in the negative electrode was 90 mol%. Operating temperature and current density were 550 °C and 200 mA cm^{-2}, respectively.

	Ca	Mg	Bi	Total
Mass / g	0.322	0.022	2.80	3.14
Density / g cm^{-3}	1.55	1.74	9.78	
Volume / cm^{3}	0.208	0.013	0.286	0.51
Atomic mass / g mol^{-1}	40.08	24.31	208.98	
Amount of substance / mol	8.03E-03	9.05E-04	1.34E-02	
Theoretical discharge capacity / Ah				0.239
Achieved discharge capacity / Ah				0.161
Achieved discharge energy / Wh				0.116
Gravimetric discharge capacity density / Ah kg^{-1}				51.3
Volumetric discharge capacity density / Ah L^{-1}				318
Gravimetric discharge energy density / Wh kg^{-1}				36.8
Volumetric discharge energy density / Wh L^{-1}				228

Supplementary Table 6 | Representative compositions of electrodes in Ca-Mg||Bi cell in Fig. 3 at partially discharged state. The initial composition of the Ca in the negative electrode was 90 mol%. The cell charge-discharged for 400 cycles at 550 °C operating temperature and at 200 mA cm^{-2} current density.

	Li (at%)	Mg (at%)	Ca (at%)	Bi (at%)	Total (at%)
Negative electrode	29.4	16.2	54.4	<0.1	100
Positive electrode	9.1	<0.001	9.27	81.6	100

Supplementary Table 7 | The metals contents of the cells.

Figure no.	*Negative electrode		**Positive electrode		**Electrolyte
	Ca (g)	Mg (g)	Bi (g)	Sb (g)	LiCl-$CaCl_2$ (g)
1a	0.397	0.964	6.00	-	13.1
1b	0.899	0.061	14.00	-	12.0
1c	1.173	0.079	-	8.19	12.5
2	0.322	0.022	2.80	-	12.0
3	1.276	0.086	14.00	-	12.0

*Determined from measured total mass of negative electrode along with stated composition of alloy.

**Measured values of mass.

Supplementary Note 1

Self-discharge current density of cells

Self-discharge current density was measured under an applied voltage of 1.25 V. Results are summarized in Supplementary Table 1. The Ca|$CaCl_2$|Bi cell at 800°C, slightly higher than melting point of $CaCl_2$ (T_m: 772°C), showed higher than 1000 mA cm^{-2} of self-discharge current density. Since the solubility of Ca metal in Ca halide salts decreases with decreasing temperature[1], the operating temperature was reduced by utilizing $CaCl_2$-

CaI_2 (T_m: 550°C, multi-anion salt, i.e. single-cation salt). The self-discharge current density of Ca|$CaCl_2$-CaI_2|Bi cell at 650°C decreased (> 300 mA cm^{-2}). By using a mixture of LiCl and $CaCl_2$ (multi-cation salt), the self-discharge current of Ca|LiCl-$CaCl_2$|Bi cell at 650°C was dramatically decreased to ≈ 10 mA cm^{-2}. To decrease the solubility of calcium, we alloyed Ca (metal A) with Mg (diluent). By alloying Ca with Mg (diluent), the self-discharge current density decreased the order of magnitude to ≈ 1 mA cm^{-2} (Ca-Mg (20-80 mol%)| LiCl-$CaCl_2$|Bi cell). The self-discharge current density was found to track with the activity of calcium in the negative electrode: the value is 2.9×10^{-2} for 20-80 mol% Ca-Mg and 1.0 for 90-10 mol% Ca-Mg at 600°C. By adding KCl or NaCl to the LiCl-$CaCl_2$ salt, the self-discharge increased from that of LiCl-$CaCl_2$ salt. This suggests that there are more complex phenomena, possibly cation-cation interactions or solubility of co-deposited metal (K and Na exhibit high solubility similar to that of Ca).

Supplementary Reference

1. Bredig, M. A., Mixtures of Metals with Molten Salts, *ORNL-3391*, 1963, Oak Ridge National Laboratory (Oak Ridge, Tennessee).
